# Investigations of new potential photo-acid generators: crystal structures of 2-[(*E*)-2-phenyl­ethen­yl]phenol (ortho­rhom­bic polymorph) and (2*E*)-3-(2-bromo­phen­yl)-2-phenyl­prop-2-enoic acid

**DOI:** 10.1107/S2056989016002942

**Published:** 2016-02-24

**Authors:** William T. A. Harrison, M. John Plater, Lee J. Yin

**Affiliations:** a Department of Chemistry, University of Aberdeen, Meston Walk, Aberdeen AB24 3UE, Scotland; b Department of Chemistry, Faculty of Science, University of Malaya, Kuala Lumpur 50603, Malaysia

**Keywords:** crystal structure, stilbene, cinnamic acid, photo-acid generator, O—H⋯π inter­actions

## Abstract

The title compounds, C_14_H_12_O, (I), and C_15_H_11_BrO_2_, (II), were prepared and characterized as part of our studies of potential new photo-acid generators. In (I), which crystallizes in the ortho­rhom­bic space group *Pca*2_1_, compared to *P*2_1_/*n* for the previously known monoclinic polymorph [Cornella & Martin (2013 ▸). *Org. Lett.* **15**, 6298–6301], the dihedral angle between the aromatic rings is 4.35 (6)° and the OH group is disordered over two sites in a 0.795 (3):0.205 (3) ratio. In the crystal of (I), mol­ecules are linked by O—H⋯π inter­actions involving both the major and minor –OH disorder components, generating [001] chains as part of the herringbone packing motif. The asymmetric unit of (II) contains two mol­ecules with similar conformations (weighted r.m.s. overlay fit = 0.183 Å). In the crystal of (II), both mol­ecules form carboxyl­ate inversion dimers linked by pairs of O—H⋯O hydrogen bonds, generating *R*_2_^2^(8) loops in each case. The dimers are linked by pairs of C—H⋯O hydrogen bonds to form [010] chains.

## Abstract 2

In the crystal of the ortho­rhom­bic polymorph of compound (I), the mol­ecules are linked into chains by O—H⋯π inter­actions. In compound (II), carb­oxy­lic acid inversion dimers are observed; the dimers are linked into chains by C—H⋯O hydrogen bonds.

## Chemical context   

Photo-acid generators can be used as additives for creating patterns in a polymer film by irradiation through a mask followed by thermal development and base treatment (Ayothi *et al.*, 2007 ▸; Kudo *et al.*, 2008 ▸; Steidl *et al.*, 2009 ▸). The UV irradiation degrades a small amount of the photo-acid generator in exposed areas, which releases a catalytic amount of a strong acid (commonly triflic acid). This acid subsequently catalyses the degradation of the *tert*-butyl­carboxyl­ate groups of a polymer film in a thermal development step, releasing carb­oxy­lic acid groups and isobutene. Treatment with base then solubilizes and removes the degraded polymer film in exposed areas, thereby creating a positive resist image (Ito *et al.*, 1994 ▸).

We are exploring new types of organic structures as potential photo-acid generators, which might offer improvements over existing substances. Scheme 1 shows how substituted *trans*-stilbenes might act as photo-acid generators *via* sequential photochemical *trans*–*cis* isomerization and ring-closing reactions. It should be noted that the photochemical cyclization of stilbenes to phenanthenes in the presence of a hydrogen acceptor such as iodine or propyl­ene oxide is well known (Mallory & Mallory, 2005 ▸). However, in the absence of an oxidant, if a leaving group is present at the ring-closure site, as in structure **3**, a rapid elimination of HX (structure **5**) might occur *via* a stabilized carbocation inter­mediate **4**. In the absence of an oxidant, the cyclized di­hydro-phenanthrene compound **6** will equilibrate back to *cis*-stilbene **2**. Stilbenes can also undergo 2π + 2π photochemical cyclo­additions (Fulton & Dunitz, 1947 ▸; Shechter *et al.*, 1963 ▸), a possible competing reaction, but the mol­ecular structures and morphology may still favour the desired reaction to proceed in a thin film.
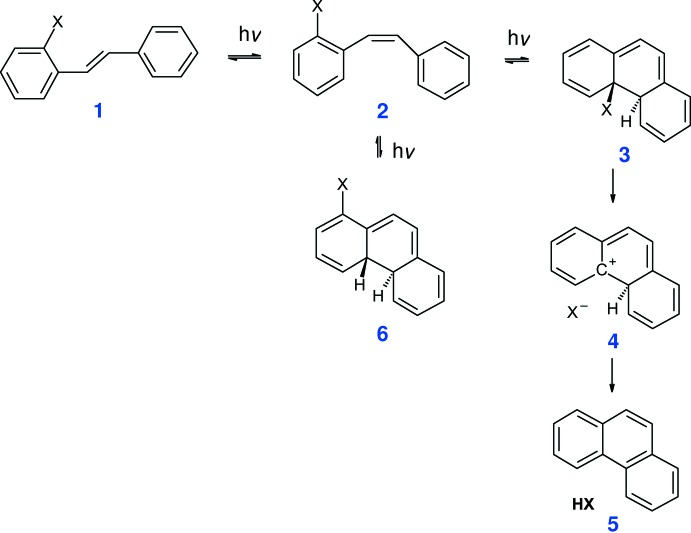


As part of these studies, the syntheses and crystal structures of the title substituted stilbenes, (I) and (II), are now described [compound (II) could also be described as a cinnamic acid derivative: the photochemical reactions of this family of compounds were reported by Schmidt (1971 ▸)]. Compound (I) is an inter­mediate in the synthesis, whereas a close analogue of compound (II) has already been shown to undergo photochemical cyclization to a phenanthrene with concomitant release of HCl (Geirsson & Kvaran, 2001 ▸). A monoclinic polymorph (space group *P*2_1_/*n*) of (I) was reported recently (Cornella & Martin, 2013 ▸) although its crystal structure was not described in detail.
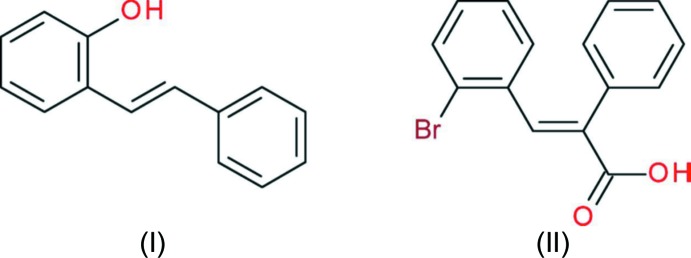


## Structural commentary   

**Figure 1:**
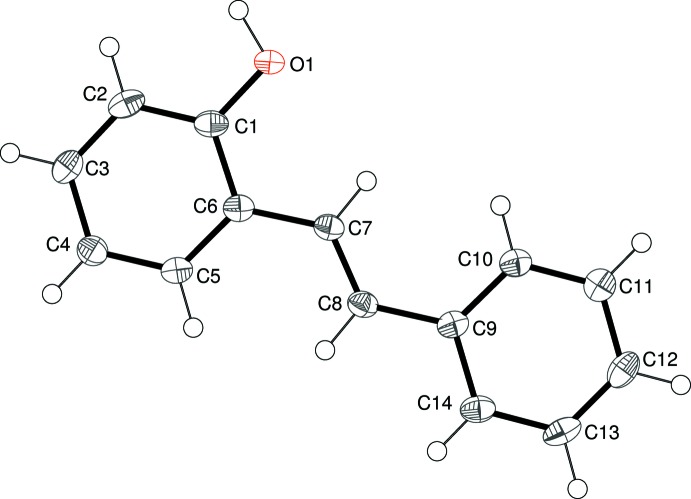
The asymmetric unit of (I), showing 50% displacement ellipsoids. Only the major disordered component for the OH group is shown (the minor component is attached to C14).

Compound (I) comprises one mol­ecule in the asymmetric unit (Fig. 1 ▸), with the –OH group disordered over two sites in a 0.795 (3):0.205 (3) ratio. For the major disorder component, the C_ar_—C_ar_—O—H (ar = aromatic) torsion angle is 172°. The mol­ecule is close to planar and the dihedral angle between the aromatic rings is 4.35 (6)°. The bond lengths of the central unit [C6—C7 = 1.4703 (19); C7—C8 = 1.3407 (16); C8—C9 = 1.4720 (18) Å] are consistent with data from previous studies of similar compounds (Tirado-Rives *et al.*, 1984 ▸; Jungk *et al.*, 1984 ▸). In the monoclinic polymorph of (I) (Cornella & Martin, 2013 ▸), the asymmetric unit consists of a half-mol­ecule, which is completed by crystallographic inversion symmetry and therefore, of course, the aromatic rings are exactly coplanar: the OH group is statistically disordered by symmetry and the corresponding C—C—O—H torsion angle for the monoclinic phase is −175°.

**Figure 2:**
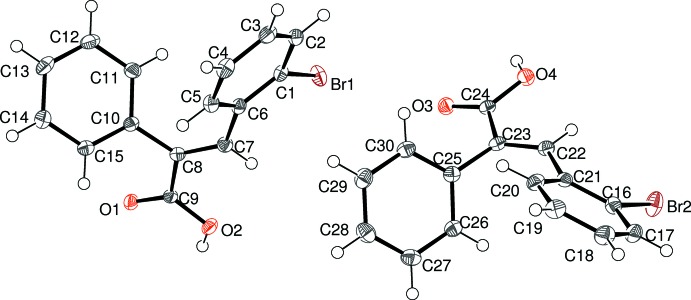
The asymmetric unit of (II), showing 50% displacement ellipsoids.

There are two mol­ecules in the asymmetric unit of (II) (Fig. 2 ▸). In the first (C1) mol­ecule, the dihedral angles between the carb­oxy­lic acid group and the phenyl and bromo­benzene rings are 61.52 (6) and 55.43 (5)°, respectively; the dihedral angle between the aromatic rings is 54.45 (5)°. The equivalent data for the second (C16) mol­ecule are 50.72 (6), 60.28 (5) and 61.48 (6)°, respectively. The C1 and C16 mol­ecules have a similar overall conformation with an r.m.s. deviation of 0.183 Å for the overlay fit for all non-hydrogen atoms. Otherwise, their bond lengths and bond angles are unexceptional and fall within the expected range of values.

## Supra­molecular features   

**Figure 3:**
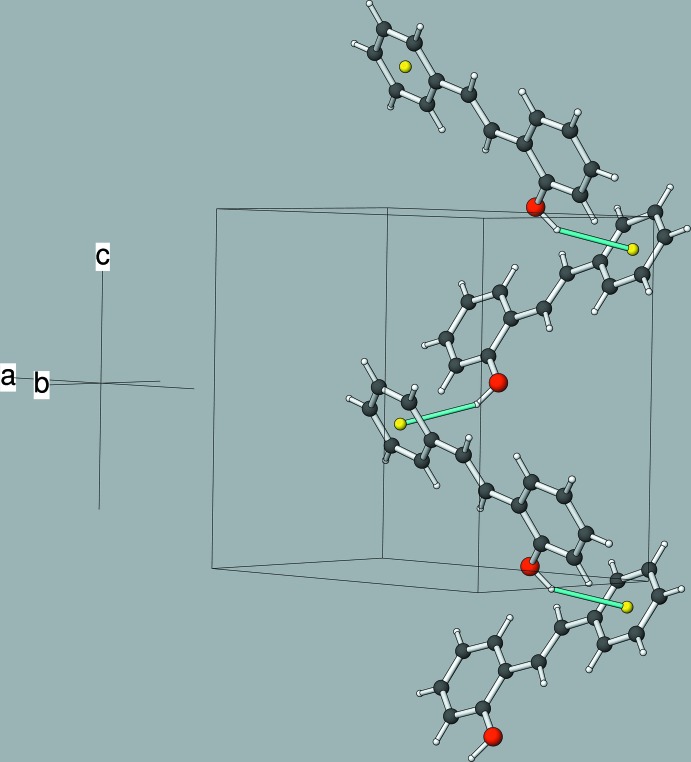
Part of a [001] chain of mol­ecules in the crystal of (I), connected by O—H⋯π inter­actions (cyan lines).

**Figure 4:**
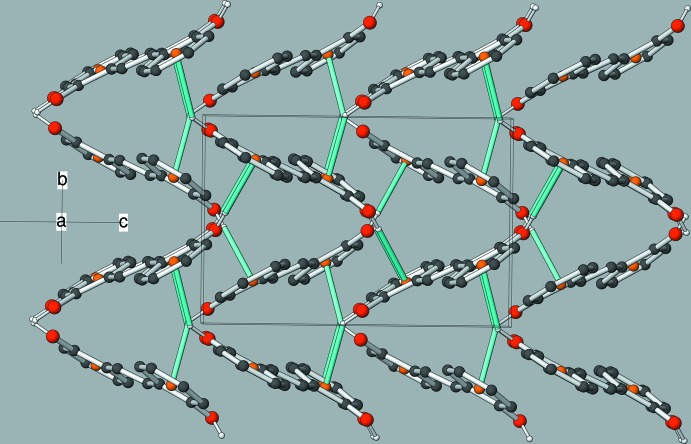
The unit-cell packing in (I), viewed approximately down [100]. The O—H⋯π inter­actions from both disordered components are shown as cyan lines.

**Figure 5:**
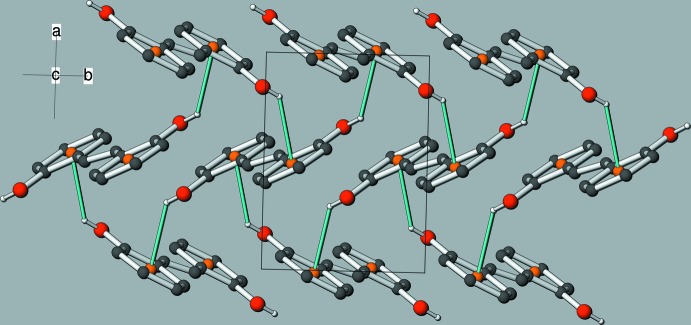
The unit-cell packing in the monoclinic polymorph of C_14_H_12_O, viewed approximately down [000] (data from Cornella & Martin, 2013 ▸). The O—H⋯π inter­actions are shown as cyan lines.

The crystal of (I) features O—H⋯π inter­actions as the main supra­molecular inter­action (Table 1 ▸). The major disorder component (O1—H1*O*) generates [001] zigzag chains, as seen in Fig. 3 ▸. The minor disorder component (O2—H2*O*) also forms [001] chains. There are also some possible very weak C—H⋯π inter­actions. The packing can be described as herringbone when viewed down [100] (Fig. 4 ▸). The monoclinic polymorph (Cornella & Martin, 2013 ▸) also features supra­molecular chains with the mol­ecules linked by O—H⋯π inter­actions but a different overall herringbone packing motif (Fig. 5 ▸).

**Figure 6:**
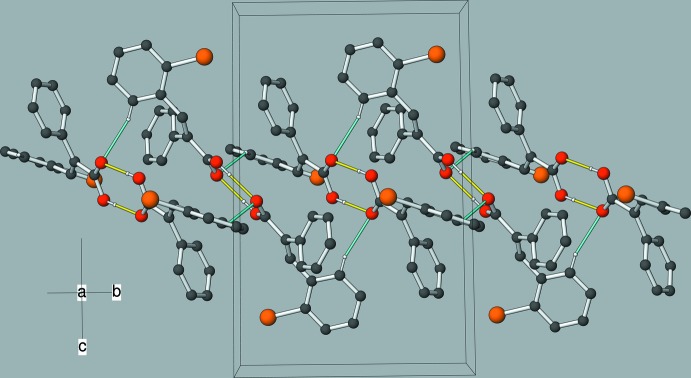
Part of a [010] chain in the crystal of (II), with O—H⋯O hydrogen bonds shown as yellow lines and C—H⋯O hydrogen bonds shown as cyan lines.

In the crystal of (II), both mol­ecules (*A* and *B*) form carb­oxy­lic acid inversion dimers linked by pairs of O—H⋯O hydrogen bonds (Table 2 ▸), which generate 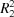
(8) loops in each case. The (*A* + *A*) and (*B* + *B*) dimers are in turn linked by pairs of C—H⋯O hydrogen bonds to generate [010] chains (Figs. 6 ▸ and 7 ▸). This hydrogen-bond scheme is ‘balanced,’ with both O1 and O3 accepting one O—H⋯O and one C—H⋯O hydrogen bond. The shortest Br⋯Br contact distance of 3.6504 (4) Å in the crystal of (II) is slightly shorter than the van der Waals radius sum of 3.70 Å for two Br atoms (Bondi, 1964 ▸).

## Database survey   

A survey of the Cambridge Structural Database (Groom & Allen, 2014 ▸) (entries updated to 22 December 2015) revealed ten crystal structures of *E*-2-hy­droxy stilbenes with different substituents including (*E*)-1,2-bis­(2-hy­droxy­phen­yl)ethene (refcode CEYKUM; Tirado-Rives *et al.*, 1984 ▸), in which the mol­ecules are linked by O—H⋯O hydrogen bonds. Two substituted *Z*-isomers are also known. A total of 28 analogues of (II) with different substituents to the aromatic rings were found in the same survey, including the parent compound, 2,3-di­phenyl­acrylic acid (refcode OJOFEZ; Fujihara *et al.*, 2011 ▸).

## Synthesis and crystallization   

Salicyl­aldehyde (0.2 g, 1.64 mmol) and benzyl­tri­phenyl­phospho­nium bromide (1.0 g, 2.31 mmol) in dry di­methyl­formamide (DMF) (30 ml) were treated with sodium methoxide powder (0.2 g, 3.70 mmol) and refluxed for 4 h (Mylona *et al.*, 1986 ▸). The reaction mixture was then cooled, acidified with dilute aqueous HCl and extracted into CH_2_Cl_2_. The organic layer was washed twice with water to remove DMF, dried over Na_2_SO_4_, concentrated *in vacuo* and purified by flash chromatography on silica gel. Hexane–diethyl ether (50:50) eluted the title compound (52 mg, 16%) as a white solid (m.p. 418–419 K), which was recrystallized from hexa­ne/diethyl ether solution to yield colourless slabs of (I); *m*/*z* 196.0886 (*M* ^+^) C_14_H_12_O requires 196.0883. UV λ_max_(CHCl_3_)/nm 230 (log ∊ 4.30), 288 (4.39) and 315 (4.40). IR (ν_max_/cm^−1^) 3528*s*, 3019*w*, 2923*w*, 2852*w*, 1585*s*, 1498*s*, 1454*s*, 1332*s*, 1249*s*, 1195*s*, 1088*s*, 974*vs*, 845*s*, 752*vs*, 724*vs*, 691*vs*, 507*vs*. ^1^H NMR (400MHz, CDCl_3_) δ 5.07 (1H, *s*), 6.79 (1H, *d*, *J* = 8.0), 6.95 (1H, *t*, *J* = 7.4), 7.14 (2H, *m*), 7.25 (1H, *t*, *J* = 6.3), 7.35 (3H, *m*), 7.52 (3H, *m*). ^13^C NMR (99.5 MHz, CDCl_3_) δ 116.1, 121.3, 123.1, 124.8, 126.7, 127.3, 127.7, 128.8, 130.3, 137.7 and 153.1 (one resonance is missing).

2-Bromo­benzaldehyde (0.5 g, 2.70 mmol) and methyl phenyl­acetate (0.6 g, 4.0 mmol) in dry DMF (30 ml) were treated with sodium methoxide powder (0.3 g, 5.6 mmol) and refluxed for 4 h. The reaction mixture was then cooled, acidified with dilute aqueous HCl and extracted into CH_2_Cl_2_. The organic layer was washed twice with water to remove DMF, dried over Na_2_SO_4_, concentrated *in vacuo* and purified by flash chromatography on silica gel. Hexane–diethyl ether (75:25) eluted (II) (65 mg, 8%) as a colourless solid, which was recrystallized from hexa­ne/diethyl ether solution as colourless rods. The starting ester was evidently hydrolysed either during the reaction or at the work-up stage; *m*/*z* 300.9866 (*M* + H) C_15_H_10_O_2_Br requires 300.9870.

## Refinement   

Crystal data, data collection and structure refinement details are summarized in Table 3 ▸. Atom H1*O* in (I) was located in a difference Fourier map and refined as riding in its as-found relative position with *U*_iso_(H) = 1.2*U*_eq_(O). The other H atoms were placed geometrically (C—H = 0.95 Å, O—H = 0.91 Å) and refined as riding atoms with *U*_iso_(H) = 1.2*U*_eq_(C,O). The O-bound H atoms in (II) were located in a difference Fourier map and refined with *U*_iso_(H) = 1.2*U*_eq_(O). The C-bound H atoms were placed geometrically (C—H = 0.95 Å) and refined as riding atoms with *U*_iso_(H) = 1.2*U*_eq_(C).

## Supplementary Material

Crystal structure: contains datablock(s) I, II, global. DOI: 10.1107/S2056989016002942/su5278sup1.cif

Supporting information file. DOI: 10.1107/S2056989016002942/su5278Isup2.cml

Supporting information file. DOI: 10.1107/S2056989016002942/su5278IIsup3.cml

CCDC references: 1454393, 1454392

Additional supporting information:  crystallographic information; 3D view; checkCIF report

## Figures and Tables

**Figure 7:**
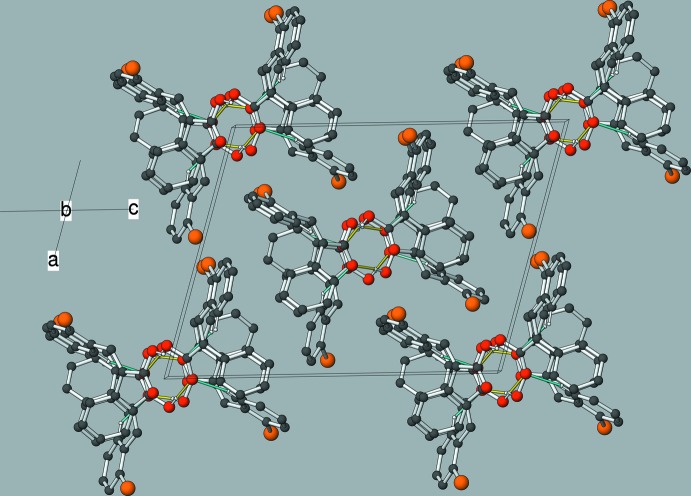
The unit-cell packing in (II), viewed approximately down [010].

**Table 1:** Hydrogen-bond geometry (Å, °) for (I) *Cg*1 and *Cg*2 are the centroids of rings C1–C6 and C9–C14, respectively.

*D*—H⋯*A*	*D*—H	H⋯*A*	*D*⋯*A*	*D*—H⋯*A*
O1—H1o⋯*Cg*2^i^	0.98	2.66	3.5028 (13)	144
O2—H2o⋯*Cg*1	0.91	2.74	3.646 (2)	179
C5—H5⋯*Cg*2^ii^	0.95	2.86	3.5337 (12)	129
C10—H10⋯*Cg*1^iii^	0.95	2.87	3.5742 (14)	132
C13—H13⋯*Cg*1^iv^	0.95	2.87	3.6015 (14)	135

Symmetry codes: (i) 
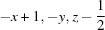
; (ii) 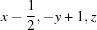; (iii) 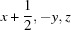; (iv) 
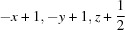
.

**Table 2:** Hydrogen-bond geometry (Å, °) for (II)

*D*—H⋯*A*	*D*—H	H⋯*A*	*D*⋯*A*	*D*—H⋯*A*
O2—H2*O*⋯O1^i^	0.84 (2)	1.80 (2)	2.6402 (16)	174 (2)
O4—H4*O*⋯O3^ii^	0.81 (2)	1.84 (2)	2.6478 (16)	178 (2)
C5—H5⋯O3^iii^	0.95	2.42	3.323 (2)	158
C20—H20⋯O1^iii^	0.95	2.52	3.3072 (19)	141

Symmetry codes: (i) 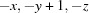; (ii) 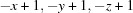; (iii) 
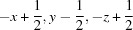
.

**Table 3:** Experimental details

	(I)	(II)
Crystal data		
Chemical formula	C_14_H_12_O	C_15_H_11_BrO_2_
*M*_r_	196.24	303.15
Crystal system, space group	Orthorhombic, *P* *c* *a*2_1_	Monoclinic, *P*2_1_/*n*
Temperature (K)	100	100
*a*, *b*, *c* (Å)	11.6193 (8), 7.6800 (5), 11.3584 (8)	13.890 (1), 10.9048 (8), 17.8121 (10)
α, β, γ (°)	90, 90, 90	90, 106.064 (1), 90
*V* (Å^3^)	1013.58 (12)	2592.6 (3)
*Z*	4	8
Radiation type	Mo *K*α	Mo *K*α
μ (mm^−1^)	0.08	3.16
Crystal size (mm)	0.27 × 0.16 × 0.04	0.19 × 0.07 × 0.07

Data collection		
Diffractometer	Rigaku CCD	Rigaku CCD
Absorption correction	–	Multi-scan (*SADABS*; Sheldrick, 2004 ▸)
*T*_min_, *T*_max_	–	0.585, 0.809
No. of measured, independent and observed [*I* > 2σ(*I*)] reflections	6984, 2271, 2132	31964, 5922, 5297
*R*_int_	0.031	0.035
(sin θ/λ)_max_ (Å^−1^)	0.649	0.650

Refinement		
*R*[*F* ^2^ > 2σ(*F* ^2^)], *wR*(*F* ^2^), *S*	0.034, 0.091, 1.06	0.025, 0.063, 1.04
No. of reflections	2271	5922
No. of parameters	146	331
No. of restraints	1	0
H-atom treatment	H-atom parameters constrained	H atoms treated by a mixture of independent and constrained refinement
Δρ_max_, Δρ_min_ (e Å^−3^)	0.19, −0.15	0.56, −0.74

Computer programs: *CrystalClear* (Rigaku, 2010 ▸), *SHELXS97* and *SHELXL97* (Sheldrick, 2008 ▸), *ORTEP-3 for Windows* (Farrugia, 2012 ▸) and *ATOMS* (Dowty, 1999 ▸).

## supplementary crystallographic information

### (I) 2-[(E)-2-Phenylethenyl]phenol Crystal data

**Table:**

C_14_H_12_O	*F*(000) = 416
*M**_r_* = 196.24	*D*_x_ = 1.286 Mg m^−^^3^
Orthorhombic, *P**c**a*2_1_	Mo *K*α radiation, λ = 0.71073 Å
Hall symbol: P 2c -2ac	Cell parameters from 7085 reflections
*a* = 11.6193 (8) Å	θ = 2.5–27.5°
*b* = 7.6800 (5) Å	µ = 0.08 mm^−^^1^
*c* = 11.3584 (8) Å	*T* = 100 K
*V* = 1013.58 (12) Å^3^	Slab, colourless
*Z* = 4	0.27 × 0.16 × 0.04 mm

### (I) 2-[(E)-2-Phenylethenyl]phenol Data collection

**Table:**

Rigaku CCD diffractometer	2132 reflections with *I* > 2σ(*I*)
Radiation source: fine-focus sealed tube	*R*_int_ = 0.031
Graphite monochromator	θ_max_ = 27.5°, θ_min_ = 2.7°
ω scans	*h* = −15→13
6984 measured reflections	*k* = −9→8
2271 independent reflections	*l* = −13→14

### (I) 2-[(E)-2-Phenylethenyl]phenol Refinement

**Table:**

Refinement on *F*^2^	Primary atom site location: structure-invariant direct methods
Least-squares matrix: full	Secondary atom site location: difference Fourier map
*R*[*F*^2^ > 2σ(*F*^2^)] = 0.034	Hydrogen site location: inferred from neighbouring sites
*wR*(*F*^2^) = 0.091	H-atom parameters constrained
*S* = 1.06	*w* = 1/[σ^2^(*F*_o_^2^) + (0.0581*P*)^2^ + 0.0566*P*] where *P* = (*F*_o_^2^ + 2*F*_c_^2^)/3
2271 reflections	(Δ/σ)_max_ < 0.001
146 parameters	Δρ_max_ = 0.19 e Å^−^^3^
1 restraint	Δρ_min_ = −0.15 e Å^−^^3^

### (I) 2-[(E)-2-Phenylethenyl]phenol Special details

**Table:**

Geometry. All e.s.d.'s (except the e.s.d. in the dihedral angle between two l.s. planes) are estimated using the full covariance matrix. The cell e.s.d.'s are taken into account individually in the estimation of e.s.d.'s in distances, angles and torsion angles; correlations between e.s.d.'s in cell parameters are only used when they are defined by crystal symmetry. An approximate (isotropic) treatment of cell e.s.d.'s is used for estimating e.s.d.'s involving l.s. planes.
Refinement. Refinement of *F*^2^ against ALL reflections. The weighted *R*-factor *wR* and goodness of fit *S* are based on *F*^2^, conventional *R*-factors *R* are based on *F*, with *F* set to zero for negative *F*^2^. The threshold expression of *F*^2^ > σ(*F*^2^) is used only for calculating *R*-factors(gt) *etc*. and is not relevant to the choice of reflections for refinement. *R*-factors based on *F*^2^ are statistically about twice as large as those based on *F*, and *R*- factors based on ALL data will be even larger.

### (I) 2-[(E)-2-Phenylethenyl]phenol Fractional atomic coordinates and isotropic or equivalent isotropic displacement parameters (Å^2^)

**Table:**

	*x*	*y*	*z*	*U*_iso_*/*U*_eq_	Occ. (<1)
C1	0.30887 (12)	0.13815 (16)	0.08810 (11)	0.0230 (3)	
H1	0.3652	0.0929	0.0386	0.028*	0.205 (3)
C2	0.19331 (12)	0.12759 (16)	0.05641 (11)	0.0258 (3)	
H2	0.1721	0.0723	−0.0152	0.031*	
C3	0.10941 (12)	0.19707 (16)	0.12852 (12)	0.0249 (3)	
H3	0.0307	0.1890	0.1067	0.030*	
C4	0.14037 (12)	0.27928 (17)	0.23349 (12)	0.0244 (3)	
H4	0.0829	0.3280	0.2831	0.029*	
C5	0.25559 (12)	0.28929 (14)	0.26490 (11)	0.0222 (3)	
H5	0.2759	0.3456	0.3364	0.027*	
C6	0.34307 (12)	0.21877 (16)	0.19426 (10)	0.0207 (3)	
C7	0.46529 (12)	0.22186 (16)	0.22775 (11)	0.0210 (3)	
H7	0.5184	0.1701	0.1746	0.025*	
C8	0.50832 (12)	0.29131 (15)	0.32669 (11)	0.0219 (3)	
H8	0.4550	0.3453	0.3786	0.026*	
C9	0.62995 (11)	0.29221 (15)	0.36292 (11)	0.0203 (3)	
C10	0.71722 (12)	0.21042 (16)	0.29798 (11)	0.0229 (3)	
H10	0.6982	0.1498	0.2277	0.027*	
C11	0.83100 (13)	0.21675 (16)	0.33495 (12)	0.0259 (3)	
H11	0.8892	0.1621	0.2893	0.031*	
C12	0.86041 (12)	0.30333 (17)	0.43927 (13)	0.0273 (3)	
H12	0.9383	0.3073	0.4646	0.033*	
C13	0.77504 (12)	0.38320 (16)	0.50516 (11)	0.0265 (3)	
H13	0.7943	0.4413	0.5763	0.032*	
C14	0.66125 (13)	0.37858 (16)	0.46739 (11)	0.0241 (3)	
H14	0.6045	0.4340	0.5121	0.029*	0.795 (3)
O1	0.39106 (10)	0.07105 (15)	0.01715 (10)	0.0261 (3)	0.795 (3)
H1O	0.3492	0.0078	−0.0443	0.031*	0.795 (3)
O2	0.5932 (4)	0.4523 (7)	0.5351 (5)	0.0305 (15)	0.205 (3)
H2O	0.6381	0.5365	0.5670	0.037*	0.205 (3)

### (I) 2-[(E)-2-Phenylethenyl]phenol Atomic displacement parameters (Å^2^)

**Table:**

	*U*^11^	*U*^22^	*U*^33^	*U*^12^	*U*^13^	*U*^23^
C1	0.0315 (7)	0.0191 (5)	0.0183 (6)	−0.0012 (5)	0.0010 (5)	0.0000 (4)
C2	0.0358 (8)	0.0207 (6)	0.0208 (6)	−0.0060 (5)	−0.0057 (5)	0.0003 (5)
C3	0.0259 (7)	0.0222 (6)	0.0266 (7)	−0.0043 (5)	−0.0061 (5)	0.0043 (5)
C4	0.0255 (7)	0.0231 (6)	0.0246 (7)	0.0004 (5)	0.0012 (5)	0.0001 (5)
C5	0.0270 (6)	0.0207 (6)	0.0190 (6)	−0.0014 (5)	−0.0005 (5)	−0.0017 (5)
C6	0.0255 (6)	0.0171 (5)	0.0194 (6)	−0.0026 (5)	0.0011 (5)	0.0005 (4)
C7	0.0245 (6)	0.0204 (6)	0.0180 (6)	−0.0004 (5)	0.0025 (5)	−0.0005 (4)
C8	0.0240 (7)	0.0207 (6)	0.0208 (6)	−0.0026 (5)	0.0045 (5)	−0.0022 (4)
C9	0.0260 (7)	0.0182 (6)	0.0166 (6)	−0.0040 (5)	−0.0001 (5)	0.0025 (4)
C10	0.0275 (7)	0.0213 (6)	0.0199 (6)	−0.0030 (5)	0.0005 (5)	0.0003 (4)
C11	0.0276 (7)	0.0233 (6)	0.0269 (7)	−0.0001 (5)	−0.0015 (5)	0.0031 (5)
C12	0.0285 (7)	0.0238 (7)	0.0296 (8)	−0.0056 (5)	−0.0102 (6)	0.0072 (5)
C13	0.0388 (8)	0.0225 (6)	0.0184 (6)	−0.0084 (5)	−0.0072 (6)	0.0017 (5)
C14	0.0330 (7)	0.0207 (6)	0.0185 (6)	−0.0038 (5)	0.0014 (5)	0.0001 (5)
O1	0.0247 (6)	0.0328 (7)	0.0209 (6)	0.0027 (5)	−0.0004 (4)	−0.0101 (5)
O2	0.027 (3)	0.035 (3)	0.030 (3)	−0.004 (2)	0.003 (2)	−0.014 (2)

### (I) 2-[(E)-2-Phenylethenyl]phenol Geometric parameters (Å, º)

**Table:**

C1—O1	1.3517 (17)	C8—H8	0.9500
C1—C2	1.3925 (18)	C9—C10	1.4024 (17)
C1—C6	1.4126 (18)	C9—C14	1.4073 (18)
C1—H1	0.9300	C10—C11	1.388 (2)
C2—C3	1.381 (2)	C10—H10	0.9500
C2—H2	0.9500	C11—C12	1.401 (2)
C3—C4	1.3963 (19)	C11—H11	0.9500
C3—H3	0.9500	C12—C13	1.386 (2)
C4—C5	1.388 (2)	C12—H12	0.9500
C4—H4	0.9500	C13—C14	1.390 (2)
C5—C6	1.4036 (18)	C13—H13	0.9500
C5—H5	0.9500	C14—O2	1.240 (5)
C6—C7	1.4703 (19)	C14—H14	0.9340
C7—C8	1.3407 (16)	O1—H1O	0.9794
C7—H7	0.9500	O2—H2O	0.9057
C8—C9	1.4720 (18)		
			
O1—C1—C2	120.33 (12)	C7—C8—H8	116.8
O1—C1—C6	118.51 (12)	C9—C8—H8	116.8
C2—C1—C6	121.17 (12)	C10—C9—C14	117.89 (12)
O1—C1—H1	0.7	C10—C9—C8	123.03 (11)
C2—C1—H1	120.0	C14—C9—C8	119.08 (12)
C6—C1—H1	118.8	C11—C10—C9	120.92 (12)
C3—C2—C1	120.33 (11)	C11—C10—H10	119.5
C3—C2—H2	119.8	C9—C10—H10	119.5
C1—C2—H2	119.8	C10—C11—C12	120.32 (13)
C2—C3—C4	119.97 (13)	C10—C11—H11	119.8
C2—C3—H3	120.0	C12—C11—H11	119.8
C4—C3—H3	120.0	C13—C12—C11	119.49 (13)
C5—C4—C3	119.55 (13)	C13—C12—H12	120.3
C5—C4—H4	120.2	C11—C12—H12	120.3
C3—C4—H4	120.2	C12—C13—C14	120.18 (12)
C4—C5—C6	122.03 (12)	C12—C13—H13	119.9
C4—C5—H5	119.0	C14—C13—H13	119.9
C6—C5—H5	119.0	O2—C14—C13	113.8 (3)
C5—C6—C1	116.96 (12)	O2—C14—C9	125.0 (3)
C5—C6—C7	123.05 (11)	C13—C14—C9	121.19 (13)
C1—C6—C7	119.98 (11)	C13—C14—H14	119.4
C8—C7—C6	125.69 (12)	C9—C14—H14	119.4
C8—C7—H7	117.2	C1—O1—H1O	105.2
C6—C7—H7	117.2	C14—O2—H2O	101.9
C7—C8—C9	126.46 (12)		
			
O1—C1—C2—C3	−179.60 (12)	C7—C8—C9—C10	−3.09 (18)
C6—C1—C2—C3	0.32 (18)	C7—C8—C9—C14	176.96 (11)
C1—C2—C3—C4	0.36 (19)	C14—C9—C10—C11	−0.88 (17)
C2—C3—C4—C5	−0.47 (19)	C8—C9—C10—C11	179.16 (12)
C3—C4—C5—C6	−0.10 (19)	C9—C10—C11—C12	0.94 (19)
C4—C5—C6—C1	0.74 (17)	C10—C11—C12—C13	−0.22 (19)
C4—C5—C6—C7	−177.62 (12)	C11—C12—C13—C14	−0.54 (19)
O1—C1—C6—C5	179.07 (11)	C12—C13—C14—O2	178.9 (3)
C2—C1—C6—C5	−0.85 (17)	C12—C13—C14—C9	0.59 (19)
O1—C1—C6—C7	−2.52 (17)	C10—C9—C14—O2	−178.0 (3)
C2—C1—C6—C7	177.56 (11)	C8—C9—C14—O2	2.0 (4)
C5—C6—C7—C8	−0.48 (19)	C10—C9—C14—C13	0.12 (18)
C1—C6—C7—C8	−178.79 (11)	C8—C9—C14—C13	−179.92 (11)
C6—C7—C8—C9	178.55 (12)		

### (I) 2-[(E)-2-Phenylethenyl]phenol Hydrogen-bond geometry (Å, º)

Cg1 and Cg2 are the centroids of rings C1–C6 and C9–C14, respectively.

**Table:**

*D*—H···*A*	*D*—H	H···*A*	*D*···*A*	*D*—H···*A*
O1—H1o···*Cg*2^i^	0.98	2.66	3.5028 (13)	144
O2—H2o···*Cg*1	0.91	2.74	3.646 (2)	179
C5—H5···*Cg*2^ii^	0.95	2.86	3.5337 (12)	129
C10—H10···*Cg*1^iii^	0.95	2.87	3.5742 (14)	132
C13—H13···*Cg*1^iv^	0.95	2.87	3.6015 (14)	135

Symmetry codes: (i) −*x*+1, −*y*, *z*−1/2; (ii) *x*−1/2, −*y*+1, *z*; (iii) *x*+1/2, −*y*, *z*; (iv) −*x*+1, −*y*+1, *z*+1/2.

### (II) (2E)-3-(2-Bromophenyl)-2-phenylprop-2-enoic acid Crystal data

**Table:**

C_15_H_11_BrO_2_	*F*(000) = 1216
*M**_r_* = 303.15	*D*_x_ = 1.553 Mg m^−^^3^
Monoclinic, *P*2_1_/*n*	Mo *K*α radiation, λ = 0.71073 Å
Hall symbol: -P 2yn	Cell parameters from 30565 reflections
*a* = 13.890 (1) Å	θ = 2.2–27.5°
*b* = 10.9048 (8) Å	µ = 3.16 mm^−^^1^
*c* = 17.8121 (10) Å	*T* = 100 K
β = 106.064 (1)°	Rod, colourless
*V* = 2592.6 (3) Å^3^	0.19 × 0.07 × 0.07 mm
*Z* = 8	

### (II) (2E)-3-(2-Bromophenyl)-2-phenylprop-2-enoic acid Data collection

**Table:**

Rigaku CCD diffractometer	5922 independent reflections
Radiation source: fine-focus sealed tube	5297 reflections with *I* > 2σ(*I*)
Graphite monochromator	*R*_int_ = 0.035
ω scans	θ_max_ = 27.5°, θ_min_ = 2.2°
Absorption correction: multi-scan (*SADABS*; Sheldrick, 2004)	*h* = −17→18
*T*_min_ = 0.585, *T*_max_ = 0.809	*k* = −13→14
31964 measured reflections	*l* = −22→23

### (II) (2E)-3-(2-Bromophenyl)-2-phenylprop-2-enoic acid Refinement

**Table:**

Refinement on *F*^2^	Primary atom site location: structure-invariant direct methods
Least-squares matrix: full	Secondary atom site location: difference Fourier map
*R*[*F*^2^ > 2σ(*F*^2^)] = 0.025	Hydrogen site location: inferred from neighbouring sites
*wR*(*F*^2^) = 0.063	H atoms treated by a mixture of independent and constrained refinement
*S* = 1.04	*w* = 1/[σ^2^(*F*_o_^2^) + (0.0335*P*)^2^ + 1.1714*P*] where *P* = (*F*_o_^2^ + 2*F*_c_^2^)/3
5922 reflections	(Δ/σ)_max_ = 0.001
331 parameters	Δρ_max_ = 0.56 e Å^−^^3^
0 restraints	Δρ_min_ = −0.74 e Å^−^^3^

### (II) (2E)-3-(2-Bromophenyl)-2-phenylprop-2-enoic acid Special details

**Table:**

Geometry. All e.s.d.'s (except the e.s.d. in the dihedral angle between two l.s. planes) are estimated using the full covariance matrix. The cell e.s.d.'s are taken into account individually in the estimation of e.s.d.'s in distances, angles and torsion angles; correlations between e.s.d.'s in cell parameters are only used when they are defined by crystal symmetry. An approximate (isotropic) treatment of cell e.s.d.'s is used for estimating e.s.d.'s involving l.s. planes.
Refinement. Refinement of *F*^2^ against ALL reflections. The weighted *R*-factor *wR* and goodness of fit *S* are based on *F*^2^, conventional *R*-factors *R* are based on *F*, with *F* set to zero for negative *F*^2^. The threshold expression of *F*^2^ > σ(*F*^2^) is used only for calculating *R*-factors(gt) *etc*. and is not relevant to the choice of reflections for refinement. *R*-factors based on *F*^2^ are statistically about twice as large as those based on *F*, and *R*- factors based on ALL data will be even larger.

### (II) (2E)-3-(2-Bromophenyl)-2-phenylprop-2-enoic acid Fractional atomic coordinates and isotropic or equivalent isotropic displacement parameters (Å^2^)

**Table:**

	*x*	*y*	*z*	*U*_iso_*/*U*_eq_	
C1	0.18672 (11)	0.20670 (14)	0.33555 (9)	0.0171 (3)	
C2	0.21064 (12)	0.11955 (16)	0.39459 (9)	0.0208 (3)	
H2	0.2476	0.1419	0.4461	0.025*	
C3	0.17974 (12)	−0.00082 (16)	0.37725 (10)	0.0228 (3)	
H3	0.1954	−0.0614	0.4171	0.027*	
C4	0.12604 (12)	−0.03303 (16)	0.30185 (10)	0.0223 (3)	
H4	0.1064	−0.1158	0.2899	0.027*	
C5	0.10111 (12)	0.05592 (15)	0.24394 (9)	0.0206 (3)	
H5	0.0632	0.0334	0.1928	0.025*	
C6	0.13072 (11)	0.17808 (15)	0.25938 (9)	0.0171 (3)	
C7	0.10814 (12)	0.27030 (14)	0.19619 (9)	0.0177 (3)	
H7	0.1615	0.3221	0.1923	0.021*	
C8	0.01866 (11)	0.28748 (14)	0.14364 (8)	0.0156 (3)	
C9	0.01101 (11)	0.37744 (14)	0.07944 (9)	0.0153 (3)	
C10	−0.07591 (11)	0.22525 (14)	0.14608 (9)	0.0158 (3)	
C11	−0.10312 (12)	0.21984 (15)	0.21603 (9)	0.0203 (3)	
H11	−0.0635	0.2605	0.2612	0.024*	
C12	−0.18786 (13)	0.15528 (16)	0.21964 (10)	0.0234 (3)	
H12	−0.2057	0.1517	0.2674	0.028*	
C13	−0.24673 (12)	0.09582 (16)	0.15388 (10)	0.0239 (3)	
H13	−0.3035	0.0497	0.1570	0.029*	
C14	−0.22209 (12)	0.10418 (15)	0.08346 (10)	0.0213 (3)	
H14	−0.2633	0.0659	0.0379	0.026*	
C15	−0.13720 (12)	0.16852 (15)	0.07977 (9)	0.0177 (3)	
H15	−0.1207	0.1739	0.0316	0.021*	
O1	−0.06993 (8)	0.41209 (10)	0.03689 (6)	0.0185 (2)	
O2	0.09775 (8)	0.41647 (11)	0.07169 (7)	0.0204 (2)	
H2O	0.0845 (15)	0.471 (2)	0.0373 (12)	0.024*	
Br1	0.230078 (13)	0.370757 (16)	0.360313 (9)	0.02454 (6)	
C16	0.88556 (12)	0.19354 (15)	0.43750 (9)	0.0194 (3)	
C17	0.94766 (12)	0.10532 (16)	0.41971 (10)	0.0234 (3)	
H17	1.0158	0.1236	0.4240	0.028*	
C18	0.90912 (13)	−0.00957 (16)	0.39570 (10)	0.0249 (4)	
H18	0.9510	−0.0708	0.3835	0.030*	
C19	0.80940 (13)	−0.03570 (15)	0.38943 (10)	0.0227 (3)	
H19	0.7834	−0.1151	0.3739	0.027*	
C20	0.74767 (12)	0.05462 (15)	0.40597 (9)	0.0204 (3)	
H20	0.6791	0.0364	0.4003	0.025*	
C21	0.78410 (11)	0.17179 (15)	0.43077 (9)	0.0173 (3)	
C22	0.72086 (12)	0.26361 (14)	0.45511 (9)	0.0179 (3)	
H22	0.7525	0.3113	0.4998	0.021*	
C23	0.62358 (12)	0.28762 (14)	0.42138 (9)	0.0169 (3)	
C24	0.57101 (12)	0.37778 (14)	0.45901 (9)	0.0172 (3)	
C25	0.56263 (12)	0.23498 (14)	0.34604 (9)	0.0173 (3)	
C26	0.59428 (12)	0.24671 (15)	0.27832 (9)	0.0203 (3)	
H26	0.6540	0.2906	0.2801	0.024*	
C27	0.53886 (13)	0.19457 (16)	0.20854 (10)	0.0234 (3)	
H27	0.5611	0.2024	0.1629	0.028*	
C28	0.45100 (14)	0.13098 (15)	0.20517 (10)	0.0249 (4)	
H28	0.4137	0.0945	0.1575	0.030*	
C29	0.41787 (13)	0.12096 (15)	0.27187 (10)	0.0239 (3)	
H29	0.3577	0.0779	0.2698	0.029*	
C30	0.47260 (12)	0.17383 (15)	0.34140 (10)	0.0201 (3)	
H30	0.4487	0.1685	0.3864	0.024*	
O3	0.48613 (8)	0.41328 (11)	0.42624 (6)	0.0207 (2)	
O4	0.62187 (9)	0.41561 (11)	0.52942 (6)	0.0207 (2)	
H4O	0.5877 (16)	0.467 (2)	0.5428 (12)	0.025*	
Br2	0.942911 (13)	0.350346 (17)	0.471076 (12)	0.03017 (6)	

### (II) (2E)-3-(2-Bromophenyl)-2-phenylprop-2-enoic acid Atomic displacement parameters (Å^2^)

**Table:**

	*U*^11^	*U*^22^	*U*^33^	*U*^12^	*U*^13^	*U*^23^
C1	0.0130 (7)	0.0176 (7)	0.0209 (7)	−0.0002 (6)	0.0049 (6)	0.0010 (6)
C2	0.0144 (7)	0.0290 (9)	0.0184 (7)	0.0024 (6)	0.0034 (6)	0.0036 (6)
C3	0.0168 (7)	0.0269 (9)	0.0254 (8)	0.0037 (6)	0.0071 (6)	0.0107 (7)
C4	0.0187 (8)	0.0179 (8)	0.0308 (9)	0.0006 (6)	0.0075 (7)	0.0030 (6)
C5	0.0175 (7)	0.0228 (8)	0.0207 (8)	0.0006 (6)	0.0039 (6)	0.0007 (6)
C6	0.0131 (7)	0.0200 (8)	0.0181 (7)	0.0014 (6)	0.0041 (6)	0.0013 (6)
C7	0.0180 (7)	0.0179 (8)	0.0176 (7)	−0.0003 (6)	0.0055 (6)	−0.0008 (6)
C8	0.0171 (7)	0.0156 (7)	0.0152 (7)	0.0002 (6)	0.0061 (6)	0.0000 (5)
C9	0.0156 (7)	0.0164 (7)	0.0147 (7)	−0.0006 (6)	0.0056 (6)	−0.0020 (5)
C10	0.0147 (7)	0.0160 (7)	0.0174 (7)	0.0027 (6)	0.0057 (6)	0.0029 (6)
C11	0.0192 (8)	0.0246 (8)	0.0171 (7)	0.0031 (6)	0.0053 (6)	0.0023 (6)
C12	0.0214 (8)	0.0298 (9)	0.0224 (8)	0.0042 (7)	0.0117 (7)	0.0068 (7)
C13	0.0184 (8)	0.0239 (9)	0.0320 (9)	−0.0010 (6)	0.0112 (7)	0.0063 (7)
C14	0.0188 (8)	0.0202 (8)	0.0247 (8)	−0.0009 (6)	0.0057 (6)	−0.0010 (6)
C15	0.0184 (7)	0.0180 (7)	0.0179 (7)	0.0013 (6)	0.0070 (6)	0.0008 (6)
O1	0.0147 (5)	0.0218 (6)	0.0185 (5)	0.0001 (4)	0.0036 (4)	0.0037 (4)
O2	0.0143 (5)	0.0249 (6)	0.0224 (6)	0.0001 (4)	0.0058 (4)	0.0086 (5)
Br1	0.02498 (9)	0.02182 (9)	0.02174 (9)	−0.00288 (6)	−0.00199 (6)	−0.00055 (6)
C16	0.0192 (7)	0.0187 (8)	0.0193 (7)	−0.0005 (6)	0.0040 (6)	0.0007 (6)
C17	0.0173 (8)	0.0286 (9)	0.0248 (8)	0.0035 (7)	0.0069 (6)	0.0019 (7)
C18	0.0243 (9)	0.0246 (9)	0.0276 (9)	0.0090 (7)	0.0100 (7)	0.0008 (7)
C19	0.0270 (8)	0.0168 (8)	0.0261 (8)	0.0010 (6)	0.0103 (7)	−0.0001 (6)
C20	0.0197 (8)	0.0213 (8)	0.0219 (8)	−0.0003 (6)	0.0085 (6)	0.0026 (6)
C21	0.0174 (7)	0.0204 (8)	0.0146 (7)	0.0019 (6)	0.0051 (6)	0.0028 (6)
C22	0.0188 (7)	0.0182 (8)	0.0177 (7)	−0.0008 (6)	0.0070 (6)	0.0002 (6)
C23	0.0187 (7)	0.0158 (7)	0.0186 (7)	−0.0005 (6)	0.0091 (6)	0.0011 (6)
C24	0.0181 (7)	0.0171 (7)	0.0180 (7)	−0.0016 (6)	0.0079 (6)	0.0015 (6)
C25	0.0184 (7)	0.0150 (7)	0.0192 (7)	0.0030 (6)	0.0063 (6)	−0.0001 (6)
C26	0.0200 (8)	0.0202 (8)	0.0222 (8)	0.0016 (6)	0.0085 (6)	0.0007 (6)
C27	0.0274 (9)	0.0240 (9)	0.0205 (8)	0.0034 (7)	0.0097 (7)	−0.0004 (6)
C28	0.0294 (9)	0.0211 (8)	0.0224 (8)	0.0012 (7)	0.0044 (7)	−0.0052 (6)
C29	0.0227 (8)	0.0196 (8)	0.0297 (9)	−0.0033 (6)	0.0077 (7)	−0.0029 (7)
C30	0.0219 (8)	0.0180 (8)	0.0228 (8)	0.0005 (6)	0.0099 (6)	−0.0003 (6)
O3	0.0174 (5)	0.0240 (6)	0.0207 (5)	0.0026 (5)	0.0052 (4)	−0.0035 (5)
O4	0.0193 (6)	0.0233 (6)	0.0195 (5)	0.0049 (5)	0.0054 (4)	−0.0045 (5)
Br2	0.02001 (9)	0.02494 (10)	0.04386 (12)	−0.00432 (6)	0.00599 (8)	−0.00679 (7)

### (II) (2E)-3-(2-Bromophenyl)-2-phenylprop-2-enoic acid Geometric parameters (Å, º)

**Table:**

C1—C2	1.388 (2)	C16—C17	1.386 (2)
C1—C6	1.400 (2)	C16—C21	1.401 (2)
C1—Br1	1.9002 (16)	C16—Br2	1.9111 (16)
C2—C3	1.389 (2)	C17—C18	1.383 (3)
C2—H2	0.9500	C17—H17	0.9500
C3—C4	1.389 (2)	C18—C19	1.388 (2)
C3—H3	0.9500	C18—H18	0.9500
C4—C5	1.388 (2)	C19—C20	1.390 (2)
C4—H4	0.9500	C19—H19	0.9500
C5—C6	1.399 (2)	C20—C21	1.400 (2)
C5—H5	0.9500	C20—H20	0.9500
C6—C7	1.477 (2)	C21—C22	1.474 (2)
C7—C8	1.346 (2)	C22—C23	1.344 (2)
C7—H7	0.9500	C22—H22	0.9500
C8—C9	1.488 (2)	C23—C25	1.489 (2)
C8—C10	1.490 (2)	C23—C24	1.490 (2)
C9—O1	1.2287 (18)	C24—O3	1.2247 (19)
C9—O2	1.3205 (18)	C24—O4	1.3236 (19)
C10—C15	1.395 (2)	C25—C30	1.399 (2)
C10—C11	1.400 (2)	C25—C26	1.399 (2)
C11—C12	1.388 (2)	C26—C27	1.390 (2)
C11—H11	0.9500	C26—H26	0.9500
C12—C13	1.390 (3)	C27—C28	1.390 (2)
C12—H12	0.9500	C27—H27	0.9500
C13—C14	1.391 (2)	C28—C29	1.392 (2)
C13—H13	0.9500	C28—H28	0.9500
C14—C15	1.389 (2)	C29—C30	1.387 (2)
C14—H14	0.9500	C29—H29	0.9500
C15—H15	0.9500	C30—H30	0.9500
O2—H2O	0.84 (2)	O4—H4O	0.81 (2)
			
C2—C1—C6	122.28 (15)	C17—C16—C21	122.53 (15)
C2—C1—Br1	118.35 (12)	C17—C16—Br2	117.43 (12)
C6—C1—Br1	119.36 (12)	C21—C16—Br2	120.03 (12)
C1—C2—C3	118.96 (15)	C18—C17—C16	119.17 (15)
C1—C2—H2	120.5	C18—C17—H17	120.4
C3—C2—H2	120.5	C16—C17—H17	120.4
C4—C3—C2	120.28 (15)	C17—C18—C19	120.21 (15)
C4—C3—H3	119.9	C17—C18—H18	119.9
C2—C3—H3	119.9	C19—C18—H18	119.9
C5—C4—C3	119.91 (16)	C18—C19—C20	119.86 (16)
C5—C4—H4	120.0	C18—C19—H19	120.1
C3—C4—H4	120.0	C20—C19—H19	120.1
C4—C5—C6	121.33 (15)	C19—C20—C21	121.55 (15)
C4—C5—H5	119.3	C19—C20—H20	119.2
C6—C5—H5	119.3	C21—C20—H20	119.2
C5—C6—C1	117.21 (14)	C20—C21—C16	116.66 (15)
C5—C6—C7	120.64 (14)	C20—C21—C22	121.33 (14)
C1—C6—C7	122.06 (14)	C16—C21—C22	121.80 (15)
C8—C7—C6	125.82 (14)	C23—C22—C21	127.45 (15)
C8—C7—H7	117.1	C23—C22—H22	116.3
C6—C7—H7	117.1	C21—C22—H22	116.3
C7—C8—C9	118.81 (14)	C22—C23—C25	125.33 (14)
C7—C8—C10	124.62 (14)	C22—C23—C24	118.99 (14)
C9—C8—C10	116.54 (13)	C25—C23—C24	115.63 (13)
O1—C9—O2	122.81 (14)	O3—C24—O4	122.96 (14)
O1—C9—C8	122.37 (13)	O3—C24—C23	121.41 (14)
O2—C9—C8	114.82 (13)	O4—C24—C23	115.63 (13)
C15—C10—C11	118.89 (14)	C30—C25—C26	118.78 (14)
C15—C10—C8	120.92 (13)	C30—C25—C23	120.85 (13)
C11—C10—C8	120.17 (14)	C26—C25—C23	120.37 (14)
C12—C11—C10	120.26 (15)	C27—C26—C25	120.34 (15)
C12—C11—H11	119.9	C27—C26—H26	119.8
C10—C11—H11	119.9	C25—C26—H26	119.8
C11—C12—C13	120.41 (15)	C26—C27—C28	120.33 (15)
C11—C12—H12	119.8	C26—C27—H27	119.8
C13—C12—H12	119.8	C28—C27—H27	119.8
C12—C13—C14	119.68 (15)	C27—C28—C29	119.73 (16)
C12—C13—H13	120.2	C27—C28—H28	120.1
C14—C13—H13	120.2	C29—C28—H28	120.1
C15—C14—C13	119.96 (16)	C30—C29—C28	120.04 (16)
C15—C14—H14	120.0	C30—C29—H29	120.0
C13—C14—H14	120.0	C28—C29—H29	120.0
C14—C15—C10	120.74 (14)	C29—C30—C25	120.73 (15)
C14—C15—H15	119.6	C29—C30—H30	119.6
C10—C15—H15	119.6	C25—C30—H30	119.6
C9—O2—H2O	106.5 (14)	C24—O4—H4O	106.9 (14)
			
C6—C1—C2—C3	1.3 (2)	C21—C16—C17—C18	−1.2 (3)
Br1—C1—C2—C3	−179.76 (12)	Br2—C16—C17—C18	179.57 (13)
C1—C2—C3—C4	0.2 (2)	C16—C17—C18—C19	0.1 (3)
C2—C3—C4—C5	−1.5 (2)	C17—C18—C19—C20	1.2 (3)
C3—C4—C5—C6	1.3 (2)	C18—C19—C20—C21	−1.5 (2)
C4—C5—C6—C1	0.2 (2)	C19—C20—C21—C16	0.4 (2)
C4—C5—C6—C7	176.79 (14)	C19—C20—C21—C22	−174.34 (15)
C2—C1—C6—C5	−1.5 (2)	C17—C16—C21—C20	0.9 (2)
Br1—C1—C6—C5	179.60 (11)	Br2—C16—C21—C20	−179.89 (11)
C2—C1—C6—C7	−178.07 (14)	C17—C16—C21—C22	175.67 (15)
Br1—C1—C6—C7	3.0 (2)	Br2—C16—C21—C22	−5.1 (2)
C5—C6—C7—C8	48.0 (2)	C20—C21—C22—C23	−41.2 (2)
C1—C6—C7—C8	−135.58 (17)	C16—C21—C22—C23	144.30 (17)
C6—C7—C8—C9	−174.75 (14)	C21—C22—C23—C25	−7.6 (3)
C6—C7—C8—C10	7.3 (2)	C21—C22—C23—C24	175.15 (14)
C7—C8—C9—O1	−168.38 (15)	C22—C23—C24—O3	171.51 (15)
C10—C8—C9—O1	9.7 (2)	C25—C23—C24—O3	−6.0 (2)
C7—C8—C9—O2	11.9 (2)	C22—C23—C24—O4	−8.7 (2)
C10—C8—C9—O2	−169.98 (13)	C25—C23—C24—O4	173.74 (13)
C7—C8—C10—C15	−131.17 (17)	C22—C23—C25—C30	125.69 (17)
C9—C8—C10—C15	50.8 (2)	C24—C23—C25—C30	−57.0 (2)
C7—C8—C10—C11	47.0 (2)	C22—C23—C25—C26	−54.8 (2)
C9—C8—C10—C11	−131.04 (15)	C24—C23—C25—C26	122.57 (16)
C15—C10—C11—C12	2.3 (2)	C30—C25—C26—C27	−2.3 (2)
C8—C10—C11—C12	−175.92 (15)	C23—C25—C26—C27	178.17 (15)
C10—C11—C12—C13	−0.3 (3)	C25—C26—C27—C28	0.5 (2)
C11—C12—C13—C14	−1.9 (3)	C26—C27—C28—C29	0.8 (3)
C12—C13—C14—C15	2.0 (3)	C27—C28—C29—C30	−0.3 (3)
C13—C14—C15—C10	0.0 (2)	C28—C29—C30—C25	−1.6 (3)
C11—C10—C15—C14	−2.1 (2)	C26—C25—C30—C29	2.9 (2)
C8—C10—C15—C14	176.04 (14)	C23—C25—C30—C29	−177.60 (15)

### (II) (2E)-3-(2-Bromophenyl)-2-phenylprop-2-enoic acid Hydrogen-bond geometry (Å, º)

**Table:**

*D*—H···*A*	*D*—H	H···*A*	*D*···*A*	*D*—H···*A*
O2—H2*O*···O1^i^	0.84 (2)	1.80 (2)	2.6402 (16)	174 (2)
O4—H4*O*···O3^ii^	0.81 (2)	1.84 (2)	2.6478 (16)	178 (2)
C5—H5···O3^iii^	0.95	2.42	3.323 (2)	158
C20—H20···O1^iii^	0.95	2.52	3.3072 (19)	141

Symmetry codes: (i) −*x*, −*y*+1, −*z*; (ii) −*x*+1, −*y*+1, −*z*+1; (iii) −*x*+1/2, *y*−1/2, −*z*+1/2.
